# Investigation of Telomerase/Telomeres system in Bone Marrow Mesenchymal Stem Cells derived from IPF and RA-UIP

**DOI:** 10.1186/1476-9255-9-27

**Published:** 2012-07-02

**Authors:** Katerina M Antoniou, George A Margaritopoulos, Athanasia Proklou, Konstantinos Karagiannis, Ismini Lasithiotaki, Giannoula Soufla, Maria Christina Kastrinaki, Demetrios A Spandidos, Helen A Papadaki, Nikos M Siafakas

**Affiliations:** 1 Department of Thoracic Medicine, Interstitial Lung Disease Unit, University Hospital of Heraklion, Crete, Greece; 2 Laboratory of Cellular and Molecular Pulmonology, Medical School, University of Crete, Crete, Greece; 3 Laboratory of Clinical Virology, Medical School, University of Crete, Heraklion, Crete, Greece; 4 Hematopoiesis Research Laboratory, Medical School, University of Crete, Crete, Greece

**Keywords:** Idiopathic Pulmonary Fibrosis, Bone Marrow Mesenchymal Stem Cells, Rheumatoid Arthritis

## Abstract

**Objective:**

Idiopathic Pulmonary Fibrosis and Rheumatoid Arthritis associated usual interstitial pneumonia seem to have the same poor outcome as there is not an effective treatment. The aim of the study is to explore the reparative ability of bone marrow mesenchymal stem cells by evaluating the system telomerase/telomeres and propose a novel therapeutic approach.

**Methods:**

BM-MSCs were studied in 6 IPF patients, 7 patients with RA-UIP and 6 healthy controls. We evaluated the telomere length as well as the mRNA expression of both components of telomerase (human telomerase reverse transcriptase, h-TERT and RNA template complementary to the telomeric loss DNA, h-TERC).

**Results:**

We found that BM-MSCs from IPF, RA-UIP cases do not present smaller telomere length than the controls (p = 0.170). There was no significant difference regarding the expression of both h-TERT and h-TERC genes between patients and healthy controls (p = 0.107 and p = 0.634 respectively).

**Conclusions:**

We demonstrated same telomere length and telomerase expression in BM-MSCs of both IPF and RA-UIP which could explain similarities in pathogenesis and prognosis. Maintenance of telomere length in these cells could have future implication in cell replacement treatment with stem cells of these devastating lung disorders.

## Introduction

Idiopathic pulmonary fibrosis (IPF) is the most devastating form of fibrosing lung diseases with a median survival of 3 years and a prognosis which is worst than that of many cancers [1,2]. The underlying pathologic pattern is that of usual interstitial pneumonia (UIP). Although recent studies have suggested that IPF is the result of repeated injuries in different sites of the lung epithelium followed by aberrant wound healing with inadequate repair of the epithelial damage, pathogenesis of IPF still remains poorly understood. Consequently and despite recent advances [3-5], there is no effective treatment currently available which can lengthen patient’s survival other than lung transplantation. Thus, the need for more effective treatment becomes imperative.

Mesenchymal stem-cells (MSCs) are one of the most intriguing novel therapeutic approaches in the field of chronic diseases [6-12] because of the ability to repair injured tissues. They possess high proliferative capacity and ability to differentiate in adipocytes, condrocytes, osteocytes, endothelial, epithelial and neuronal cells depending on the culture conditions [13]. Moreover, there are data suggesting that bone marrow (BM)-MSCs have the ability to differentiate and function as airway and parenchymal lung cells [14]. It has been shown in animal model of bleomycin (BLM)-induced fibrosis that (BM)-MSCs express several chemokine receptors such as CXCR4 which ligand, CXCL12, is induced in murine lungs [15-17] suggesting that bone marrow stem cells could be recruited and mobilized to the injured lung through a CXCR4 dependent mechanism. Recently we have shown that CXCR4 is overexpressed in BM-MSCs of patients with IPF [18] suggesting that the abovementioned scenario could also be applied to humans.

Telomeres are repeated DNA sequences acting as protective caps for chromosomes. Telomere shortening is one of the molecular mechanisms underlying ageing and critically short telomeres trigger chromosome senescence and lead to cell death [19]. Telomerase is a specialized polymerase that adds telomere repeats to chromosomes compensating the telomere shortening and consists of two components: a catalytic component, telomerase reverse transcriptase (h-TERT) and an RNA component (h-TERC) [20].

The aim of our research was to investigate the reparative ability of BM-MSCs in patients with IPF by evaluating telomerase expression and telomere length and propose a novel therapeutic approach for this dismal disease. In our research we have also included patients with rheumatoid arthritis (RA) as in this disease, when there is interstitial lung involvement and unlike the rest of collagen tissue disorders, the most predominant pathologic pattern is UIP and in this case prognosis is similar to IPF [21]. We aimed to prove that telomere length and telomerase expression are not different in patients group compared to healthy controls proposing the possibility of cell replacement treatment as a novel therapeutic approach.

## Patients

We have studied prospectively 6 patients with IPF, 7 patients with RA-UIP whose characteristics are shown in Table 1. Patients were recruited from the Interstitial Lung Disease Unit (ILDU) at the Department of Thoracic Medicine of Heraklion. The control group included 6 subjects, age-matched with the patients who underwent posterior iliac crest aspirate because of suspected hematologic malignancies. We have included in our research those with negative biopsy results and thus considered as healthy subjects. These patients have been studied retrospectively and we did not have details other than age and gender in order to be matched with the patients.

**Table 1:** Clinical characteristics of the patients studied

**UPN**	**Age/sex**	**Disease**	**Duration of lung disease (months)**	**Medication at presentation**	**FVC/DLCO**
1	77/M	RA-UIP	0	No therapy	75.3/47.2
2	71/F	RA-UIP	12	PDN/AZA/NAC	68.7/22.1
3	75/F	RA-UIP	0	No therapy	89.7/34.8
4	72/M	RA-UIP	0	MTX-LEFLUNEMIDE*	119.3/68.4
5	76/F	RA-UIP	6	PDN/AZA/NAC	82.8/60.1
6	50/F	RA-UIP	12	PDN/AZA/NAC	94.5/49.6
7	81/F	RA-UIP	0	No therapy	76.5/32
8	74/M	IPF	0	No therapy	70/70
9	78/M	IPF	0	No therapy	41.6/25
10	82/M	IPF	0	No therapy	75.6/38.5
11	85/M	IPF	0	No therapy	88/31
12	70/M	IPF	0	No therapy	83/58
13	72/M	IPF	0	No therapy	75/45

Abbreviations: PDN: prednisolone, MTX: methotrexate, AZA: azathioprine, NAC: N-acetyl-cysteine, UPN: unique patient number.

* The patient was on treatment for Rheumatoid Arthritis.

The diagnosis of IPF was made in 3 cases by surgical biopsy and the histologic diagnosis of Usual Interstitial Pneumonia (UIP) was obtained. In the remaining 3 cases the diagnosis was made according to the recently published ATS/ERS guidelines [1].

The diagnosis of RA-UIP was made in 2 cases by surgical biopsy and the histologic diagnosis of UIP was obtained. In the remaining 5 cases, patients had a “definite RA-UIP” pattern based on HRCT criteria, with basilar predominant reticulation, traction bronchiectasis and honeycombing, with limited ground-glass opacities [22].

Diagnosis of RA was based on clinical criteria in accordance with the international societies guidelines [23].

Ethical Committee of the University of Crete has approved the study and all participants (patients and control subjects) were informed on the scope of the study and gave their written informed consent [18,24].

## Methods

### BM-MSCs in vitro expansion and differentiaition

BM mononuclear cells (BMMCs) obtained from posterior iliac crest aspirates were cultured in Dulbecco’s Modified Eagle Medium-Low Glucose (DMEM-LG; Gibco/Invitrogen, Paisley, Scotland)/10% fetal calf serum (FCS; Hyclone, Logan-Utah,USA)/100 IU/ml Peniciline-Streptomycin (MSC medium) and MSCs were grown and flow cytometric analysis of MSCs in each passage was performed as previously described [25,26]. Upon reaching confluency at passage 2 (P2) trypsinized MSCs were centrifuged and after performing a cell count in a Neubauer haemocytometer at least 1.000.000 cells were used for further processing.

MSCs from P2 were induced for differentiation. Adipogenic differentiation was induced following 21-day culture of cells in MSC medium supplemented with 10%FCS/0.5 mM 1-methyl-3-butylisoxanthine/1 μM dexamethasone (Dex)/0.2 μM indomethacin/10 μg/ml insulin and adipogenesis was assessed by Oil Red O staining. Osteogenic differentiation was induced following 21-day culture of cells in MSC medium supplemented with 0.1 μM Dex/0.15 mM ascorbate-2-phosphate/3 mM NaH_2_PO_4_ and osteogenesis was assessed by alkaline phosphatase (ALP)/von Kossa staining. For chondrogenic induction, MSCs were pelleted in 15 ml tubes and cultured for 21 days in DMEM-High Glucose (Gibco), supplemented with 6.25 μg/ml insulin/6.25 μg/ml transferring/1.33 μg/ml linoleic acid/1.25 mg/ml bovine serum albumin/1 mM sodium pyruvate/0.17 mM ascorbate-2-phosphate/0.1 μM Dex/0.35 mM L-proline/6.25 ng/ml selenous acid/0.01 μg/ml transforming growth factor-β1 (R&D Systems) and chondrogenesis was assessed by Alcian blue stain. All reagents were purchased from Sigma unless otherwise indicated.

### BM-MSCs immunophenotypic characteristics

Trypsinised MSCs from P2 were immunophenotypically characterised by flow-cytometry, using anti-CD29 (4B4; Cyto-Stat, Beckman-Coulter, Fullerton, California, USA), anti-CD44 (J173; Immunotech/Coulter, Marseille, France), anti-CD73 (AD2; Pharmingen, San Diego, California, USA), anti-CD90 (F15.42; Immunotech/Coulter), anti-CD105 (SN6; Caltag, Burlingame, California, USA), anti-CD146 (P1H12; Pharmingen), anti-CD45 (IMMU19.2; Immunotech/Coulter), anti-CD14 (RMO52; Immunotech/Coulter) and anti-CD34 (QBend10; Beckman-Coulter) monoclonal antibodies.

### Real-time reverse transcriptase-polymerase chain reaction assay

MSCs at P2 were homogenized in the TRIzol® reagent (Invitrogen, Carlsband, CA), total RNA was extracted and cDNA synthesized by reverse transcription (RT) with the Thermoscript™ RT kit (Invitrogen). Genes mRNA expression was measured using a real-time RT-PCR assay with SYBR-Green I. β-actin was used as the internal control, in order to normalize h-TERT and h-TERC expression levels. The mRNA-specific primers used are listed in Table 2.

**Table 2:** Primer sequences used for quantitative Real-time RT-PCR

***Gene***	***Primer pair Sequence (5′-3′)***	***Annealing Temperature***
***hTERT***	FOR: TGACACCTCACCTCACCCAC	51°C
	REV: CACTGTCTTCCGCAAGTTCAC	
***hTERC***	FOR: GCCTGCCGCCTTCCACCGTTCATT	59°C
	REV: GACTCGCTCCGTTCCTCTTCCTG	
***TELOMERE***	FOR: CGGTTTGTTTGGGTTTGGGTTTGGGTTTGGGTTTGGGTT	56°C
	REV: GGCTTGCCTTACCCTTACCCTTACCCTTACCCTTACCCT	
***ACTIN***	FOR: CGGCATCGTCACCAACTG	58°C
	REV: GGCACACGCAGCTCATTG	

### Telomere length measurement

The relative telomere length was estimated by real-time PCR as described originally by Cawthon [27], with minor modifications. β-Globin (HBG) was used as control single-copy-gene. Primer sequences are listed in Table 3. Genomic DNA was extracted from P2 MSCs using TRIzol_ reagent (Invitrogen, Carlsband, CA). 35 ng of DNA were used for the reactions in a final volume of 20 μl. iTaq SYBR Green Supermix with ROX (Biorad, Hercules,CA) was used for the PCR reactions. The concentrations of reagents for telomere PCRs were 1X iTaq SYBR Green Supermix, 100nM forward, and 900nM reverse primers. For HBG reactions 400nM were used from each primer. For telomere PCRs, 40 cycles were conducted: 15 s at 95 °C and 1 m at 56 °C. For HBG PCRs, 40 cycles were performed: 15 s at 95 °C and 1 m at 58 °C. PCRs were carried out in Mx3000P Real-Time PCR system (Stratagene, La Jolla, CA, USA). All reactions were performed in duplicates. The relative telomere length was reflected by the T/S ratio, where T is the amount of telomere (T = 2^-Ct telomere^) and S is the amount of the single-copy-gene (S = 2^-Ct HBG^): T/S = 2^-Ct telomere^/2^-Ct HBG^ = 2^-(Ct telomere-Ct HBG)^ = 2^-ΔCt^

**Table 3:** Primer sequences used for relative telomere length estimation with RT-PCR

	**Forward**	**Reverse**
**Telomere**	5^′^CGGTTTGTTTGGGTTTGGGTTTGGGTTTGGGTTTGGGTT-3^′^	5^′^GGCTTGCCTTACCCTTACCCTTACCCTTACCCTTACCCT-3^′^
**HBG**	5^′^GCTTCTGACACAACTGTGTTCACTAGC-3^′^	5^′^-CACCAACTTCATCCACGTTCACC-3^′^

### Statistical analysis

Differences in relative telomere length and m-RNA expression of h-TERC and h-TERT genes between patients group and controls have been tested with Kruskal-Wallis test and a value of p < 0.05 has been considered significant

## Results

### MSCs immunophenotype and differentiation potential

Immunophenotypic analysis of MSCs from all groups of patients and healthy controls at the end of P2 demonstrated that cultures constituted of a homogenous cell population positive for CD73, CD90, CD146, CD105, CD29, CD44 and negative for CD45 and CD34 surface antigens. P2 MSCs were able to differentiate towards the adipogenic, osteogenic and chondrogenic lineages in healthy individuals, as well as in all groups of patients.

### Telomerase expression and telomere length

**Figure 1:**
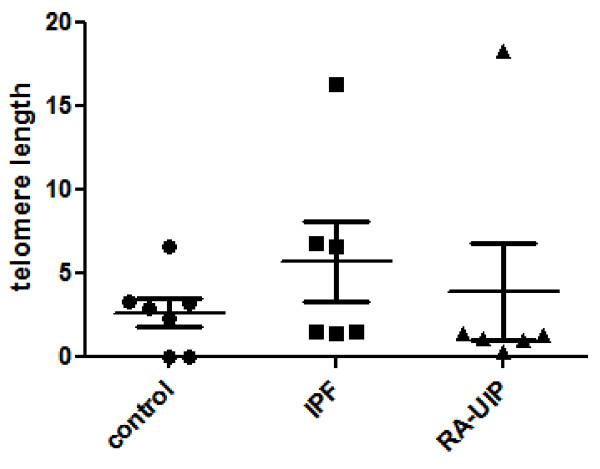
Relative telomere length in the three study groups.

We found that the relative telomere length in both patients group (IPF and RA-UIP) did not differ compared to healthy controls (p = 0.17). This finding suggests that BM-MSCs obtained from patients group are able to maintain telomere length and therefore could be proposed as possible replacement treatment (Table 4 and Figure 1).

**Table 4:** Relative telomere length in the three study groups

	**RA-UIP**	**IPF**	**Controls**	**p-value**
**Telomere length**	0.663(1.476, 11.548)	1.118 (0.747, 9.867)	1.876 (0.038, 3.221)	0.170

Comparison was performed with Kruskall Wallis H test. Values are presented as median (interquartile ranges).

Both h-TERT and h-TERC genes were expressed at the m-RNA level in all patients’ group and control subjects. However, we did not observe any statistically significant difference in gene expression of h-TERT and h-TERC between study groups (p = 0.107 and p = 0.634 respectively) (Table 5).

**Table 5:** mRNA expression of hTERT and hTERC in the three study groups

	**RA-UIP**	**IPF**	**Controls**	**p-value**
**hTERT**	0.50 (0.00, 1.30)	1.45 (0.78, 21.37)	0.50 (0.27, 0.77)	0.107
**hTERC**	0.10 (0.00, 3.60)	0.05 (0.00, 6.78)	0.20 (0.08,0.20)	0.634

Comparison was performed with Kruskall Wallis H test. Values are presented as median (interquartile ranges).

## Discussion

It is well known that IPF is the result of multiple injuries in different sites of lung epithelium followed by inadequate repair characterised by the migration of resident fibroblasts, BM progenitors of fibroblasts (fibrocytes) and fibroblasts derived from a process called epithelial-mesenchymal transition (EMT), formation of fibroblast and myofibroblast foci and exaggerated production of extracellular matrix (ECM). The subgroup of patients with RA associated interstitial lung disease who present with the underlying pathologic pattern of UIP has the same prognosis with IPF [22] suggesting the possibility of shared pathogenetic pathways. For both diseases there is still no effective treatment other than lung transplantation. Cell replacement treatment with MSCs is promising because of the proliferation capacity and ability of these cells to repair injured tissues.

Telomeres shorten successively with each cell division and when they achieve a critical length, activate a p53-dependent mechanism that leads to apoptosis or replicative senescence [28]. Short telomeres are expected to compromise the replicative potential of progenitor cells that remain in tissues after injury. We aimed to show that BM-MSCs from patients with IPF and RA-UIP are able to maintain telomeres length despite the high proliferation capacity and thus maintain the reparative ability. Our most important finding is that BM-MCSs from patients present relative telomere length which did not differ compared to healthy controls and thus could be used for autologous transplant. Moreover, they express, although weakly, both genes of telomerase at the same level with healthy controls suggesting a plausible mechanism for the maintenance of telomere length. The findings of the current study are not similar with previous findings of our group [29]. However, it should be stressed that these were preliminary findings of our work and patients included in that cohort were older, had more severe disease (DLCo < 30%), with few of them on ambulatory oxygen, than the patients included in the current cohort. In addition, it was previously observed that 25% of sporadic cases with IPF had shorter telomeres in peripheral leukocytes without coding mutations in telomerase [30] and we believe that this scenario could also be applied to BM-MSCs explaining the discrepancy of the findings between the two studies.

Recent studies have shown that MSCs may abrogate fibrosis, but unfortunately in most of them, an animal model of BLM-induced fibrosis has been used and as it is known, this model does not represent the progressive and lethal nature of IPF. Nonetheless, MSCs seem to partially abolish lung injury in animal models of fibrosis, emphysema and inflammatory lung injury and participate in organ regeneration [31]. Interestingly, lung engraftment of MSCs administered systematically occurs at low levels in normal mice whereas it is increased in injured murine lung after exposure to BLM. MSCs adopt an epithelial-like phenotype and have a beneficial effect by reducing inflammation, collagen deposition and metalloproteinases activation within lung tissue [32]. Additionally, in another study it was observed that prominin-1/CD133(+) epithelial progenitor cells expanded from adult mouse lung and of bone marrow origin, are able to express both stem and haematopoietic cell markers and differentiate in vitro into type II epithelial cells. When intratracheally administered in mice treated with BLM, they engrafted into the lungs, differentiated into type II epithelial cells and suppressed proinflammatory and profibrotic gene expression protecting form the development of pulmonary fibrosis [33]. Alternatively to BM-MSCs, embryonic stem cells (ESCs) which present similar ability to differentiate in any cell of the body have been studied as replacement treatment. Alveolar epithelial type II cells derived from human ESCs were transplanted in the lungs of mouse treated with BLM and differentiated into type I alveolar epithelial cells abrogating the inflammatory and fibrotic response [34].

The system telomerase/telomere has been implicated in the pathogenesis of IPF. It was observed that mutations in both components of telomerase are present in 8-15% of patients with familial IPF [35,36]. On the other hand, mutations in the essential telomerase genes are also present in 1-3% of sporadic cases [30,37]. Plainly mutations lead to loss of telomerase function and to telomere shortening. Interestingly though, it was observed that in sporadic cases of IPF telomeres are short in lymphocytes, granulocytes and alveolar epithelial cells compared to age-matched controls even in absence of telomerase mutations [30,37].

Considering the fact that BM-MSCs have high mitotic activity and go through a large number of replication, one may expect that they express high levels of telomerase in order to prevent telomere shortening. Surprisingly and in accordance with our findings, it was observed that adult stem cells express low levels of telomerase and telomerase activity is low [38,39]. This may be a defensive mechanism against malignant transformation as it was shown that adult stem cells play an important role in cancer development and maintenance [40-43]. Plainly, there may be a mechanism of telomere maintenance other than telomerase. It is suggested that the alternative lengthening of telomeres pathway, a recombination based DNA replication mechanism, may maintain telomere length [44,45]. In addition, it was observed that subtelomeric hypomethylation facilitates telomere elongation in mammalian cells suggesting that such epigenetic modification of cromatin may occur also in MSCs [46]. On the other hand, even a low telomerase expression and activity are required for both replication and differentiation as it is shown that MSCs from a telomerase activity knocked-down mouse failed to differentiate into adipocytes or condrocytes and that telomerase overexpressing human MSCs have enhanced in vivo bone formation potential [47,48].

In conclusion, we have shown that BM-MSCs from patients with IPF and RA-UIP maintain the same telomere length with healthy donors suggesting the possible use of these cells in cell replacement treatment for both diseases. The same treatment has been also proposed for other chronic diseases such as amyotrophic lateral sclerosis (AML) [49] as it was found that human BM-MSCs from patients present the same telomere length with healthy donors. Plainly, caution is recommended as there are a lot of issues that need to be clarified such as whether the level of lung engraftment is sufficient for regenerative purposes, the likelihood of cell rejection, and the possibility of causing local damage i.e. favouring the development of fibrosis or lung neoplasms [50]. Definitely, further studies are needed before start using stem cells safely for pulmonary diseases.

## Competing interests

The author(s) declare that they have no competing interests.

## Authors’ contribution

KMA and GAM designed the study, performed the statistical analysis and wrote the manuscript. AP and KK contributed to patients recruitment and evaluation. IL and GS carried out the RT-PCRs. MCK and HAP obtained the posterior iliac crest aspirates. DAS and NMS coordinated the study and helped to draft the manuscript. All read and approved the final version of the manuscript.
